# Network based systems biology approach to identify diseasome and comorbidity associations of Systemic Sclerosis with cancers

**DOI:** 10.1016/j.heliyon.2022.e08892

**Published:** 2022-02-08

**Authors:** Md Khairul Islam, Md. Habibur Rahman, Md Rakibul Islam, Md Zahidul Islam, Md Mainul Islam Mamun, A.K.M. Azad, Mohammad Ali Moni

**Affiliations:** a Dept. of Information & Communication Technology, Islamic University, Kushtia-7003, Bangladesh; b Dept. of Computer Science & Engineering, Islamic University, Kushtia-7003, Bangladesh; c Department of Applied Physics and Electronic Engineering, University of Rajshahi, Rajshahi-6205, Bangladesh; d Artificial Intelligence and Cybernetics Lab, Department of Computer Science and Engineering, The Independent University Bangladesh, Dhaka 1229, Bangladesh; e Artificial Intelligence & Digital Health Data Science, School of Health and Rehabilitation Sciences, Faculty of Health and Behavioural Sciences, The University of Queensland, St Lucia, QLD 4072, Australia; f Computer Science & Engineering, Pabna University of Science, Pabna 6600, Bangladesh

**Keywords:** Bioinformatics, Comorbidities, Associations, Gene, Gene set enrichment analysis, Correlation, Gene ontology, Systemic Sclerosis, Pathways, Cancer

## Abstract

Systemic Sclerosis (SSc) is an autoimmune disease associated with changes in the skin's structure in which the immune system attacks the body. A recent meta-analysis has reported a high incidence of cancer prognosis including lung cancer (LC), leukemia (LK), and lymphoma (LP) in patients with SSc as comorbidity but its underlying mechanistic details are yet to be revealed. To address this research gap, bioinformatics methodologies were developed to explore the comorbidity interactions between a pair of diseases. Firstly, appropriate gene expression datasets from different repositories on SSc and its comorbidities were collected. Then the interconnection between SSc and its cancer comorbidities was identified by applying the developed pipelines. The pipeline was designed as a generic workflow to demonstrate a premise comorbid condition that integrate regarding gene expression data, tissue/organ meta-data, Gene Ontology (GO), Molecular pathways, and other online resources, and analyze them with Gene Set Enrichment Analysis (GSEA), Pathway enrichment and Semantic Similarity (SS). The pipeline was implemented in R and can be accessed through our Github repository: https://github.com/hiddenntreasure/comorbidity. Our result suggests that SSc and its cancer comorbidities share differentially expressed genes, functional terms (gene ontology), and pathways. The findings have led to a better understanding of disease pathways and our developed methodologies may be applied to any set of diseases for finding any association between them. This research may be used by physicians, researchers, biologists, and others.

## 1 Introduction

Scleroderma, or Systemic Sclerosis (SSc), is an autoimmune rheumatic sickness portrayed by extreme creation and amassing of collagen, called fibrosis, in the skin, inner organs as well as injuries to small arteries. SSc is very heterogeneous and generally affects patients' lungs, heart, and kidney that cause most of the deaths.

There are many studies claimed that there might be a possibility of developing cancer in SSc patients [1]. There are three autoantibodies associated with SSc namely: anti-centromere, anti-RNA polymerase III (RNAP III), and anti-Scl-70. Many previous experiments have shown that the existence of anti-RNAP polymerase III in patients appears to have a near spatial association with cancer initiation and SSc initiation [2][3]. The relationship between SSc and Cancer is more complicated than anyone suspected and thus resulted in several different pathways [4]. According to the European Scleroderma Trials and Research group's (EUSTAR Database) report, cancer causes almost 11% of death in SSc patients. Therefore, Cancer guides to the third most mortality in the case of SSc diagnosed patients [5]. Another research claimed that patients with SSc are at risk of being diagnosed with Cancer within SSc patients' first year. Moreover, immuno-suppressants therapies are used to cure this autoimmune disorder may also contribute to the establishment of cancer [6][7].

Interestingly, the presence and extent of SSc diffuse lung disease (scarring of lung tissue in internal organs like Interstitial Lung Disease) have indeed been reported as a potential cause for Lung cancer in previous research [8]. It is suspected that SSc patients mostly diagnosed with lung disease [9][10] at a young age as well as women [11] have a greater chance of developing rapidly growing lung malignancy. In an Italian study, Lung cancer was found approximately in 5 percent of SSc patients (16 out of 318 patients). Furthermore, SSc patients have a 4.2-times chance of growing Lung cancer relative to ordinary people [12]. In another study, Lung cancer and Lymphoma have the largest rates of occurrence [13] in SSc patients. Lymphoma incidence was observed in SSc patients, with a standardized incidence ratio (SIR) of 2.1 [14] in Sweden. Over 56 years old SSc patients (130 people) have been observed, while 66 patients diagnosed with Lymphoma (mostly in B-cell tumors) and 28 patients diagnosed with Leukaemia (in Lymphocytes) [15][16].

The distinctive evidence of specific co-occurring disorders in a person referred to as a comorbid condition—has become a typical practice because of its extraordinary alert on the forthcoming diseases and also reduce medical services cost [17]. Therefore, the accessibility of adaptable, simple to utilize programming structures, called bioinformatics, are fundamental to assist the investigation of comorbid condition of a pair of disease, over a enormous transcriptomic datasets of the patient's different organs or cells [18]. In recent bioinformatics approach, researcher tried to establish mechanisms to find shared genes, molecular pathways, protein-protein interactions, diseasome network, cluster analysis and semantic similarity in terms of DEGs and GO pathways, that help in determining a comorbid condition between a pair of disease [19][20]. Previously researchers applied computational modelling to predict a comorbid condition and clinical bioinformatics approaches to validate comorbidities [21]. Since we have discovered common significant genes, molecular pathways, semantic similarity and other risk factors between SSc and cancer (pair like SSc vs Lung, SSc vs Lymphoma, and SSc vs Leukaemia), as well as SSc is a heterogeneous disease, so it is much more difficult to understand common molecular mechanisms and therapeutic drug targets for SSc with coexisting diseases.

To avoid such complexity in research area, diagnosing diseases, or medicine discovering, we have developed a bioinformatics pipeline for SSc and its cancer comorbidities. The approach demonstrate the shared genes, molecular pathways of SSc and a cancer (Lung, Lymphoma, and Leukaemia). As there is no usual way of determining comorbid condition, we have followed previous research and implement few additional bioinformatics mechanisms together to develope a pipeline. Our proposed pipeline, combined bioinformatics approaches like analysing transcriptomics, pathways, protein-protein interactions and identification of hub proteins along with semantic similarity, performed much better than previous clinical data based bioinformatics approaches as well as the recent discovered individual mechanism. In this regard, we have collected 7 different transcriptomic datasets of the selected diseases. Then we have performed gene expression profiling of the transcriptomic data that used to identify significant biomarker genes and regarding gene ontology, and molecular pathways. Identified significant genes also utilised to conduct gene set enrichment analysis, semantic similarity, and cluster network. The findings assisted to prove that SSc established the risk of being diagnosed with cancer disease [22]. To summarise, our proposed methodology proved that SSc is highly associated with Lung Cancer than Leukaemia or Lymphoma. In fact, We may apply that methodology to any omics (transcriptomic) datasets including RNA-seq or microarray data. Anyone with a simple understanding of programming and biological knowledge may use the proposed pipelines to validate comorbid conditions between pairs of diseases.

## 2 Methods

### 2.1 Overview of available data

In this study, RNA-seq (high throughput sequencing) datasets were collected from the public repository, freely accessible, including the National Biotechnology Information Center (NCBI) [23] and BioJupies [24]. Atleast 6 samples were considered from the raw datasets to maximize the full strength of this analysis. The factors considered in choosing the datasets for such analysis are as follows.

1. In our analysis, we have excluded duplicate samples (based on GSM IDs) that are present across different datasets;
2. SSc and cancers have many datasets but we consider only those datasets which have both control and case samples;
3. Just human data was included for the study while non-human dataset was avoided;
4. We have counted the number of significant genes from Differentially Expressed Genes (DEGs) considering two conditions: absolute log fold change value is greater than or equal to 1 as well as the adjusted p-value ≤0.05.

For the quest of the highly expressed genes in SSc patients, RNA-seq datasets were collected from GEO repositories and included for our analysis: GSE102864, a review of 9 control samples and 9 SSc subjects from Dermal fibroblast (skin) tissue [25]; GSE104174, a review of 15 control samples and 15 SSc subjects from monocytes, lymphocytes (blood) tissue [26]. To prove our hypothesis we also collected selected cancer's RNA-seq dataset from GEO repositories. For lung cancer our included datasets: GSE60052, a review of 7 control samples and 7 small cell lung cancer subjects from lung tissue [27]; GSE99531, a review of 4 control samples and 4 non small cell lung cancer subjects from T cell (blood) tissue [28]. For leukaemia cancer our included datasets: GSE94453, a review of 5 control samples and 4 leukaemia cancer subjects from Acute Myeloid Leukemia (AML) cell (blood) tissue [29]; GSE107071, a review of 3 control samples and 3 leukaemia cancer subjects from AML cell (blood) tissue [29]. For Lymphoma cancer our included dataset: GSE106092, a review of 2 control samples and 4 Mantle Cell Lymphoma (MCL) cancer subjects from mantle cell line (blood) tissue [30]. MCL is a type of non-Hodgkin lymphoma.

### 2.2 Gene set enrichment analysis

Gene Set Enrichment Analysis (GSEA) is used for interpreting gene expression data as well as functionally enriched GO terms on the different conditions or disease states. This approach works with multiple genes that share a similar biological process, chromosome position, or modulation, and thereby reveals several similar biological pathways, which facilitates the biological interpretations of the findings [31]. The GSEA approach analysis information at the standard of gene sets that utilized prior biological knowledge, such as gene pathways, gene expression profiles [32]. Our identified genes were associated with different genotypes (phenotypes) [33]. While GSEA takes nearly all the genes into account in the analysis, not just those above a randomly chosen threshold in terms of log2 fold change. We have implemented the GSEA using package: ‘topGO’ [34]. Firstly, we have got the most specific GOs corresponding to the set of genes. By implementing ‘topGO’ package, we have got the total number of GO and their interactions among them for our regarding significant genes of each dataset. At last, we have got top genes annotated to the GO terms. On the found data, we have applied Fisher's Exact Test to find gene counts accountable for enriching particular GO terms and Kolmogorov-Smirnov (KS) tests to compare continuous distributions.

### 2.3 Pathway enrichment analysis

In many bioinformatics approaches, it is very important to measure the significance of the overlap between a given set of genes of interest and previously annotated sets of genes to identify functional pathways that are associated with these genes set [35]. The advent of high-throughput experimental technologies such as microarrays and RNA sequencing have revolutionized molecular biology's knowledge [36][37][33]. Analysing the most significant genes against a database of well-annotated gene sets, such as molecular pathways, allows us to see that a gene set is functionally important or not [38] [39][40]. We have used KEGG repositories to identify DEG-enriched molecular pathways for inferring molecular pathways relevant to SSc that also interact with the cancer diseases [41]. KEGG is an interactive database resource for researching the associations of the biological systems with cells and organisms using molecular-level information which make the way in developing the disease in a patient. The networks of KO nodes, the KEGG pathway maps, KEGG modules, and BRITE hierarchies are formed, which reflecting high-level of cells and organisms functions [42].

### 2.4 Ontology-based semantic similarity estimation

Gene Ontology (GO) has an enormous community public database that gives a lot of controlled vocabularies (biological or biochemical terms), represents gene products depending on their features in the cell. [43]. It is a community-oriented database of gene ontologies to help organic annotation of significant genes [44][45]. In the hierarchical ontology graph, terminology placed closely together (i.e. with a few intermediate terminologies between them) appear to be ontologically more equivalent than any others far apart [46]. Various methods were used to quickly access all aspects of the data accommodated for the information and to allow valuable comprehension of exploratory knowledge using the GO, for example by enrichment analysis [44][45].

Semantic similarity methods have been used to provide a framework for their pragmatic analysis using GO [47]. Ontologies were presented as directed acyclic graphs (DAGs) in which the term is represented as nodes and relations as edges. Semantic associations with each of its predecessor terms were identified by the DAG (a graph of ontology's subset). To summarise semantic similarity tries to find similarities between two terms (genes, GO) [48].

A GO term R can generally be defined as DAG_R_ = (R,T_R_,E_R_) where T_R_ is the DAG_R_ set of GO term (such as R and each of its predecessor terms in the GO graph) and E_R_ is the DAG_R_ set of all edges (semantic relationships). The semantic value of R is:

(1) $$\left\{ \begin{array}{cc} S_{R}(R)=S_{R}(t)=1 & \text{t=R}\\ S_{R}(t)=max\{w_{e}⁎S_{T}(t^{\prime })|t^{\prime }\in children\;of(t) & \text{t ≠ T} \end{array}\right.$$

where w_e_ is the edge e's ($e\in T_{R}$) semantic contribution multiplier, the standard term t with its child term $t^{\prime }$. According to the form of connection, the semantic contribution is allocated between 0 and 1. For R, the global semantic meaning is determined as:

(2) $$SV(R)=\sum S_{R}(t)$$

and the semantic similarity between two terms is:

(3) $$sim(R,K)=\frac{\sum_{t\in T_{R}\cap T_{K}}(S_{R}(t)+S_{K}(t))}{SV(R)+SV(K)}$$

Given two sets of terms

$$R_{1}=\{t_{11},t_{12},...t_{1m}\}$$

$$K_{1}=\{t_{21},t_{22},...t_{2n}\}$$

The length of the very first set of terms is n, and the length of the second set of terms is m. For two given sets, we used the best-match average (BMA) to measure the semantic similarity as follows:

(4) $$sim_{BMA}=\frac{\sum {}_{i=1}^{n}max{}_{1\leq j\leq m}\{t{}_{1i},t{}_{2j}\}+\sum {}_{j=1}^{m}max{}_{1\leq i\leq n}\{t{}_{1i},t{}_{2j}\}}{n+m}$$

with i, j indices on R, K terms.

### 2.5 Pipeline overview

**Figure 1:**
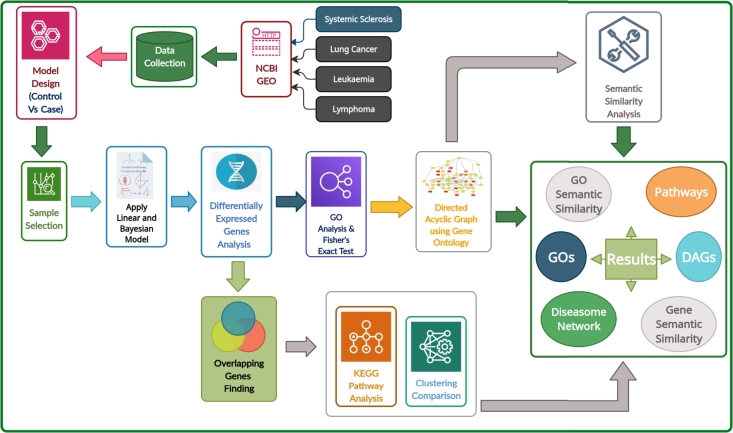
Schematic diagram of our proposed workflow. Here we have selected disease datasets from NCBI Gene expression Omnibus. Then, we have developed our case-control pairs of the selecting samples to identify the Differentially Expressed Genes (DEGs). Then significant genes were determined based on two criteria from DEGs: adjusted p-value ≤0.05 and |*logFC*| ≥ 1. GO, KEGG pathways, and Semantic Similarity analysis were performed on the DEGs to reveal SSc and its cancer comorbidities.

The schematic diagram of the proposed workflow is showed in Fig. 1. The developed bioinformatics pipeline was implemented using the R language, available for public access at: https://github.com/hiddenntreasure/comorbidity. At first, we have considered transcriptomic datasets from Gene expression omnibu's (GEO) repositories for the selected diseases. Both SSc and cancer's high throughput sequencing (RNA-seq) samples (GSM records) were collected considering the fact: diseases-affected samples and healthy controls samples.

Before selecting GSM records of a particular dataset, the case-control pairs (Like: SSc vs Lung cancer) were designed according to the research aim. The designed case-control pairs for SSc, were SSc-control vs SSc-affected and for Cancers (Lymphoma, Leukaemia, and Lung cancer)-affected vs control. GSM samples were investigated for better understanding of control and case samples. ‘BioJupies’ [24], a online platform, is used for GSM records selection as well as differential expression analysis of genes. In some cases, BioJupies can automatically select healthy-control and case samples. If not then we select manually reviewing the details of GSM records. A Bayesian approach is applied for filtering candidate genes according to the designed case-control pair using two parameters, they are adjusted P-value (using Benjamini & Hochberg's FDR correction method and the cut-off p-value is 0.05) and log2 fold change (logFC) [49]. We used ‘topGOdata’, a R packages, on the significant genes to determine the GO term along with the annotation for mapping. Then conduct Fisher's Exact Test to filter GO terminology and find relationships within genes [34]. We calculated semantic equivalence for all immunological pathologies after obtaining the enriched GO terms corresponding to the SSc and cancer datasets by using *mgoSim* function available in *GoSemSim* package [50]. We used the KEGG pathways database to understand the common molecular pathways between SSc and cancer diseases, a manually created mapping which is accessible by anyone interested in diseases relationships [41]. Shared significant genes have been compared against the KEGG database to identify and demonstrate molecular mechanisms among the diseases.

At the end of our methodology we have got the statistical summary of the datasets, genes-GO term, semantic similarity matrix (and a tree diagram) using genes and GO terminology, DAG, and KEGG molecular pathways. Alongside the most common pathway associated with the chosen pathologies, we have constructed a genomic network using common DEGs that were highly expressed in SSc and any of the cancer.

The pipeline was constructed using the following packages of Bioconductor, ‘genefilter’ [51]: is a method for extracting genes from previous research with high-throughput data; ‘topGO’ [34]: this package offers metrics to evaluate GO terms when estimating for the GO graph topology. It may also possible to introduce and apply various statistical tests and different approaches to remove local correlations and incompatibilities between suggested GO terms.; ‘GOSemSim’ [50][52]: computation of semantic similarities among GO, groups of GO, regulatory genes and gene categories; ‘clusterProfiler’ [53]: for the KEGG pathways simulation analysis.

### 2.6 Protein-protein interaction (PPI) analysis

After identifying DEGs, we have used the DEGs for protein-protein interactions. To show the protein-protein interaction which reveals the hub proteins. We used STRING to create protein interaction networks for the DEGs found in our enrichment study. Since the number of DEGs was low, we used a medium trust score (500) to build the PPI to identify hub proteins.

## 3 Results

### 3.1 Summary results

Table 1 shows the SSc and its cancer comorbiditie's statistical summary based on our methodological needs. Here in this study, we have collected two datasets for each disease from GEO database except only one dataset considered for Lymphoma. Table 1 includes GEO accession numbers for each dataset, cell descriptions, the number of case and control samples, raw genes, significant gene, raw GSEA and Fisher GSEA.

**Table 1:** Summary of preliminary results.

Disease	GEO	Cell	Case	Control	Raw	Significant	Raw	Fisher
type	Accession	Source	Samples	Samples	Genes	Genes	GSEA	GSEA
SSc	GSE102864	Dermal fibroblasts	9	9	12346	1955	10431	5408
	GSE104174	Monocyte derived	16	15	14406	393	11798	4120
		macrophages						

Lung Cancer	GSE99531	CD8+ T cells	10	4	18151	2928	13549	6283
	GSE60052	flash	10	7	16546	2870	13229	6333

Lymphoma	GSE106092	Mantle cell	4	2	14060	835	11021	5517
		lymphoma cell						

leukaemia	GSE107071	Acute myeloid	3	3	11995	139	9880	3428
		leukaemia cell-line						
	GSE94453	Acute myeloid leukaemia	4	4	10305	636	8772	4345

At first, we have conducted differential expression analysis on the count data of each dataset considering the case-vs-control samples. Thus we got candidate genes for each datasets which shown as raw genes in the Table 1. Then two conditions were applied on the candidate genes to identify significant/biomarker genes. This helped us to achieve dimensionally reduced downstream analyses via focusing only on the active part of the biological system. To that extent, we have considered $Log_{2}$ transformed expression fold changes (FC), where a threshold value of 1 for the absolute $Log_{2}FC$ was considered.

As well as, we have conducted empirical Bayes procedure, where the significance cut-off p-value < 0.05 was considered. Thus acquired significant or biomarker genes in downstream analyses showed in the Table 1 as seventh feature.

The initial step of the GO enrichment analysis is to discover raw GSEA. Hence, we performed gene-ontology mapping using DEGs and GO term from the biologic process (BP). Feature 8th showed in Table 1, depict the number of nodes/annotated genes to the Gene Ontology term. Fisher's Exact Test is a measurable test depends mainly on the possibility tables to contemplate the criticalness of the relationship between two sets of characterizations. This is also used to classify statistically important and enriched biological roles. For example, the functional annotations of a group of genes can be compared with the rest of the genome, or the functional profile, since two experimental conditions can be compared against each other. The 9th features in Table 1 show the number of important GO terminology enriched in the Fisher Exact test.

Taking the top 150 DEGs, Table 2 shows the number of up-regulated and down-regulated genes for all the datasets. Then we compare SSc and its cancers comorbidities datasets considering only the top 150 DEGs. After that, we find the common significant genes between SSc and its cancers associations: EDNRB, ALDH2, CXCL2, SMTN, C1QB, CD93, JDP2, COL8A2, NAV3, PKIB, RNPC3, SMOX.

**Table 2:** A description of DEGs achieved from various organs of the human body using the approach suggested.

Disease	Number of samples	Organ/Tissue	Selected dataset	DEG up	DEG down
SSc	110	Dermal fibroblasts	GSE104174	74	76
SSc	110	monocyte derived macrophages	GSE102864	58	92
LC	380	CD8+Tcells	GSE99531	95	55
LC	380	flash	GSE60052	97	53
Lp	262	mantle cell lymphoma cell	GSE106092	79	71
Lk	1212	acute myeloid leukaemia cell line	GSE107071	52	19
Lk	1212	acute myeloid leukaemia	GSE94453	99	51

**Figure 2:**
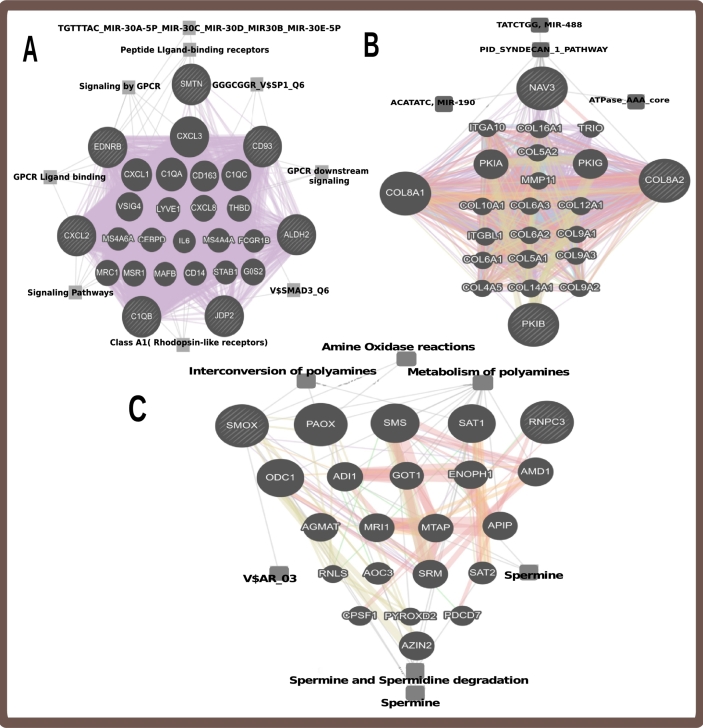
Cluster Network of enriched pathways of shared DEGs (differentially expressed genes) between Systemic Sclerosis and cancer. The most significant pathways that were associated with the selected pathologies and their network percentile coverage included. A) Pathways between SSc and LC are: *Peptide ligand-binding receptors* (0.74%), *Class A1 (Rhodopsin-like receptors)* (0.29%), *GPCR ligand binding* (0.29%), *GPCR downstream signaling* (0.13%), *Collagen biosynthesis and modifying* (10.15%), *Assembly of collagen fibrils* (7.14%), *Metabolism of polyamines* (5.34%), *Amine Oxidase reactions* (0.46%), and *Spermine and spermidine degradation* (0.38%). B) Pathways between SSc and Lk are: *ATPase AAA core* (1.1%), *PID SYNDECAN 1 PATHWAY* (0.54%), *ACATATC, MIR-190* (0.19%) and *TATCTGG, MIR-488* (0.15%), *Metabolism of polyamines* (2.27%). C) Pathways between SSc and Lp are: *spermine and spermidine degradation* (0.38%), *Interconversion of polyamines* (0.06%), *Amine Oxidase reactions* (0.0028%), *Spermine* (0.13%) and *V$AR* 03 (0.063%).

We also use the Genemania, a online tool, to build a cluster network with similar genes between SSc and any of the selected cancer [54]. Fig. 2A, 2B and 2C show the cluster network, based on the shared genes, delivered from Genemania that represents the enriched pathways between SSc and Lung Cancer, SSc and Leukaemia, SSc and Lymphoma, respectively.

Firstly, the most prominent pathways between SSc and LC were associated with the selected pathologies and their network percentile coverage: Peptide ligand-binding receptors (0.74%), Class A1 (Rhodopsin-like receptors) (0.29%), GPCR ligand binding (0.29%), GPCR downstream signaling (0.13%), Collagen biosynthesis and modifying (10.15%), Assembly of collagen fibrils (7.14%), Metabolism of polyamines (5.34%), Amine Oxidase reactions (0.46%), Spermine and spermidine degradation (0.38%) etc. Secondly, the most prominent pathways between SSc and Lk are: ATPase AAA core (1.1%), PID SYNDECAN 1 PATHWAY (0.54%), ACATATC, MIR-190 (0.19%) and TATCTGG, MIR-488 (0.15%) etc. Thirdly, the most prominent pathways between SSc and Lp are: Metabolism of polyamines (2.27%), spermine and spermidine degradation (0.38%), Interconversion of polyamines (0.06%), Amine Oxidase reactions (0.0028%), Spermine (0.13%) and V$AR 03 (0.063%) etc.

**Figure 3:**
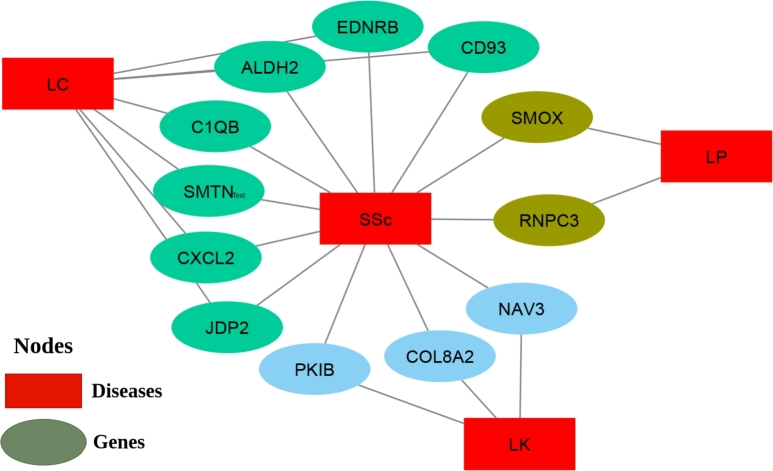
Identified and representing the common genes between systemic sclerosis and different types of cancer using diseasome network (Disease-gene association network). This network helps to check whether co-morbidities genes are in the vicinity of SSc genes. The network illustrates that there were 8 genes shared between SSc and Lung Cancer, 3 genes shared between SSc and Leukaemia and 2 genes shared between SSc and Lymphoma.

In Fig. 3, shows the significant associations among these cancers with the effects of Systemic Sclerosis, disease-genes association-ship networks were constructed for DEGs using Cytoscape plugins41, centered on SSc. ‘JDP2’, ‘EDNRB’, ‘CD93’, ‘ALDH2’, ‘C1QB’, ‘SMTN’, and ‘CXCL2’ were common between SSC and LC. ‘RNPC3’ and ‘SMOX’ were common between SSc and LP. ‘COL8A2’, ‘PKIB’, and ‘NAV3’ were common between SSc vs Lk.

### 3.2 Pathway enrichment analysis of DEGs reveals shared biological functions between SSc and its cancer comorbidities

**Figure 4:**
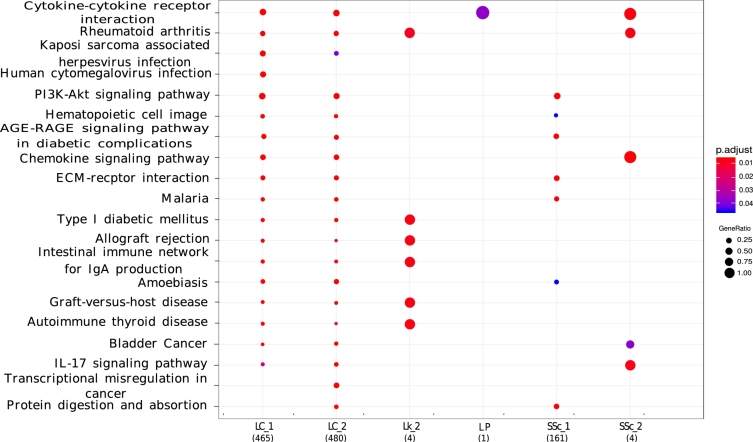
A dot plot illustrates to demonstrate the pathway Enrichment of the differentially expressed genes using KEGG pathway database. Here the dot corresponds to the significantly enriched pathways, colour and size of each dot refers to the significance of the enrichment test (adjusted p-value < 0.05) and the ratio of overlapping DEGs with that of a particular KEGG pathway, respectively. KEGG's most commonly enriched pathways between SSc and considered cancer were : Cytokine-cytokine receptor interaction, Rheumatoid arthritis, Pl3K-Akt signaling pathway, Hematopoietic cell lineage, AGE-RAGE signaling pathway in diabetic complications, Chemokine signaling pathway, ECM-receptor interaction, Malaria, Amoebiasis, Graft-versus-host disease, Autoimmune thyroid disease, Bladder cancer, IL-17 signaling pathway, and protein digestion and absorption.

Pathway enrichment test is a tool for observing the functional relevance of a group of genes/proteins/molecules by measuring the significance of their overlap with an annotated group of genes/proteins/molecules known *apriori* for their particular biological function, namely pathways analysis. We have hypothesized that such pathway enrichment test would reveal shared biological functions between SSc and its comorbidities with cancer disease via observing commonly enriched pathways against DEGs. KEGG pathways is an known database that mapped pathway with annotation. An over-representation statistical test of the DEGs, namely Fisher's exact test, has been conducted with adjusted p-values < 0.05 to obtain significantly enriched pathways. As shown in Fig. 4, we have found that there were several commonly enriched KEGG pathways between SSc (at least one) and considered cancer diseases including Cytokine-cytokine receptor interaction, Rheumatoid arthritis, Pl3K-Akt signaling pathway, Hematopoietic cell lineage, AGE-RAGE signaling pathway in diabetic complications, Chemokine signaling pathway, ECM-receptor interaction, Malaria, Amoebiasis, Graft-versus-host disease, Autoimmune thyroid disease, Bladder cancer, IL-17 signaling pathway, and protein digestion and absorption.

### 3.3 GO enrichment and DAG of genetic interrelationship from genomics data

**Figure 5:**
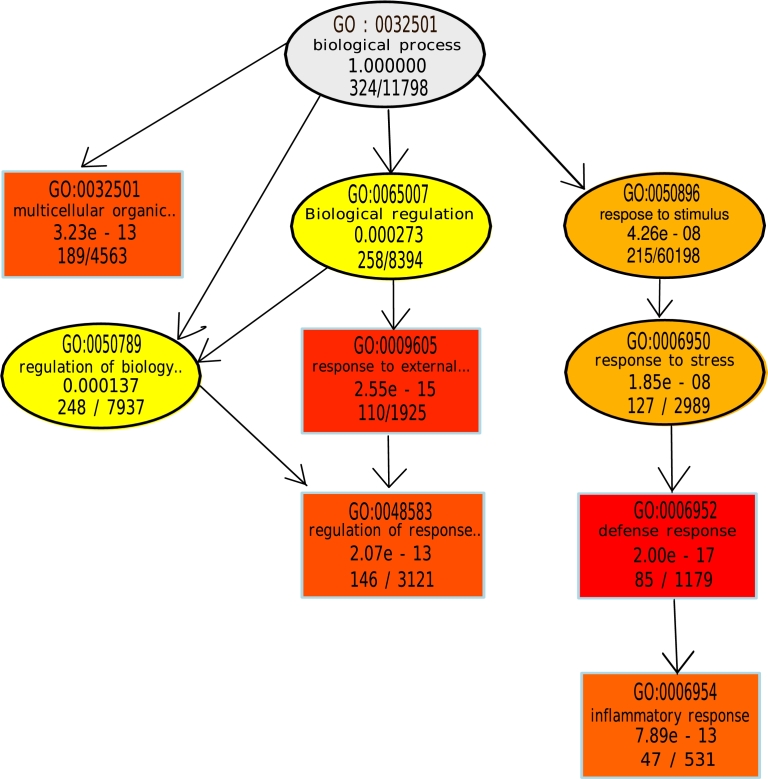
A directed acyclic graph (DAG), subgraph of the GO terms, induced by the enriched GO terms (from GSEA) on the GSE104174 dataset. And the 5 most significant terms were represented by the rectangles shape while the colour intensity proportionally increased with their level of significance. Each node of GO terms contains some basic information to portray the enrichment along with a disease, i.e. term ID, term name (trimmed), enrichment p-value, and the ratio of overlapping DEGs. Top 5 GO terms for the GSE104174 dataset includes a multicellular organismal process (GO:0032501), response to the external stimulus (GO:0009605), regulation of response to the stimulus (GO:0048583), defense response (GO:0006952), and inflammatory response (GO:0006954).

We've found many DEGs from RNA-seq data using two conditions (p-value and logFC), yet their biological interpretation hard to discover as well as perplexing. Therefore, the enrichment analysis of DEGs was conducted to identify functional GO terms that are strongly enriched using known gene set annotations. The ‘topGO’, a R package, was used to perform GO enrichment analysis using Fisher's Exact test as well as Kolmogorov-Smirnov on each dataset. And also generate hierarchical Direct Acyclic Graphs (DAG) using the most enriched GO terms corresponding to the datasets. Fig. 5 shows the DAG of significant GO terms regarding the GSE104174-dataset, where GO terminology represents the spectrum of the GO mappings with the five most important GO terminology. DAG is created using the *elim* algorithm in ‘topGO’ R package [34]. As shown in Fig. 5, the top 5 GO terms for the GSE104174 dataset includes: a multicellular organismal process (GO:0032501), response to the external stimulus (GO:0009605), regulation of response to the stimulus (GO:0048583), defense response (GO:0006952), and inflammatory response (GO:0006954).

Fig. 5 is used to show the important interrelations (top 5 in this case) of GO terms throughout the hierarchical structure. However, it also implies that all GO terminology is not essential to acknowledge the biological meaning of a disease. The following list of GO were common terms found between SSc and Lung cancer [34]:

• GO:0040011: locomotion
• GO:0006955: immune response
• GO:0006928: cellular component movement
• GO:0022610: biological adhesion
• GO:0007155: cell adhesion
• GO:0032501: multicellular organismal process
• GO:0030198: extracellular matrix organization
• GO:0016477: cell migration
• GO:0048870: cell motility
• GO:0051674: localisation of cell
• GO:0048856: development of an anatomical
• GO:0007275: multicellular organism development
• GO:0009653: anatomical structure morphogenesis
• GO:0032502: developmental process
• GO:0030154: cell differentiation
• GO:0051239: regulation of multicellular organismal
• GO:0048731: system development
• GO:0048646: anatomical structure formation involved in morphogenesis
• GO:0048869: cellular developmental process structure

The following list of GO were common terms found between SSc and Leukaemia:

• GO:0034612: response to tumor necrosis factor
• GO:0006955: immune response
• GO:0022610: biological adhesion
• GO:0007155: cell adhesion
• GO:0030155: regulation of cell adhesion

The following list of GO were common terms found between SSc and Lymphoma:

• GO:0032501: multicellular organismal process
• GO:0048856: anatomical structure development
• GO:0007275: multicellular organism development
• GO:0032502: developmental process

### 3.4 Semantic similarity of enriched GO terms shows comorbidities of SSc with other cancers

**Figure 6:**
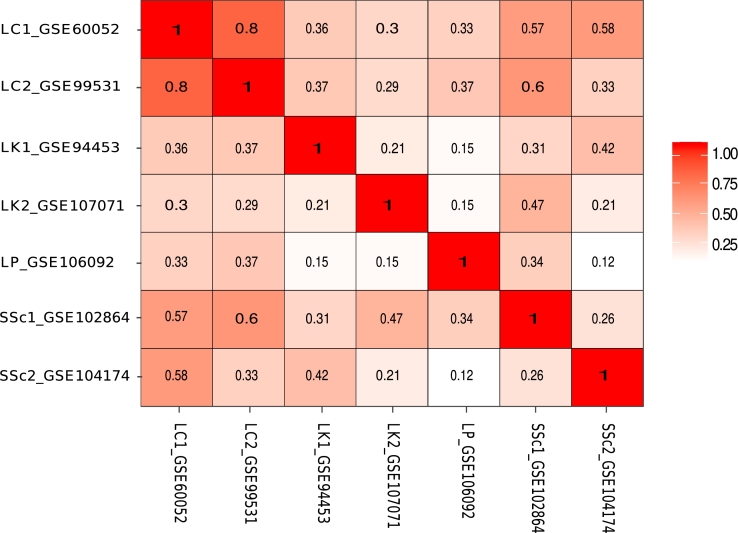
The matrix of pair-wise semantic similarities of sets of all GO terms enriched with DEGs across different datasets. The matrix demonstrated using legend (depicting the score of semantic similarity) and accession number of the disorder. It implies that Lung cancer is associated with SSc (accession no: GSE102864) with score 0.57 (accession no: GSE60052) and 0.6 (accession no: GSE99531) and also SSc (accession no: GSE104174) with score 0.58 (accession no: GSE60052).

**Figure 7:**
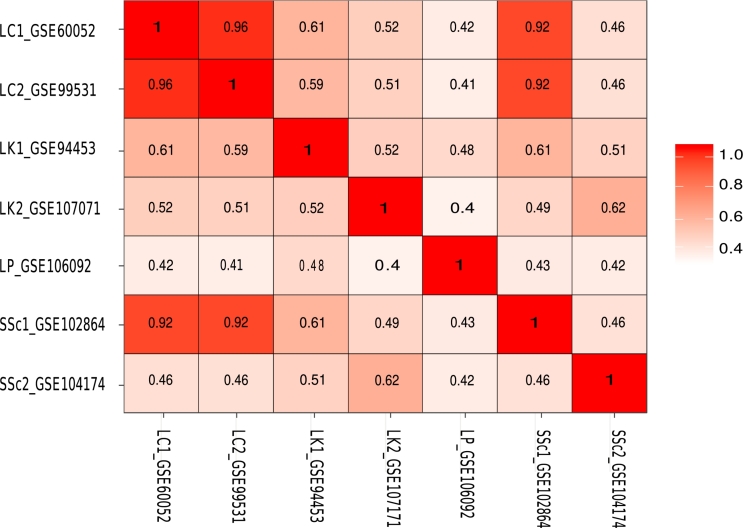
The semantic similarity matrix is based on the top 5 terms of GO. The matrix demonstrated using legend (depicting the score of semantic similarity) and accession number of the disorder. It reveals that Lung Cancer highly associated with SSc (accession no: GSE102864) with score 0.92 (accession no: GSE99531 and GSE60052). Also discovered that SSc (accession no: GSE102864) associated with Leukaemia with a score of 0.61 (accession no: GSE94453), additionally SSc (accession no: GSE104174) with a score of 0.62 (accession no: GSE107071).

To reveal SSc and its cancer's comorbid condition, we had focused on the shared significant genes, molecular pathways, enrichment analysis. Which disclosed biological functions and their rationale in developing cancer in SSc patients. We have also conducted a semantic similarity experiment with enriched GO terms across different datasets. Hence, the semantic similarity matrix specifies the likeliness of co-occurring two disease, though we have identified the association of cancer disease in SSc patients. Using mgoSim function in ‘GoSemSim’ R package [see Methods], it has been observed that SSc shares moderately its biological functions with only Lung cancer when considered all the enriched GO terms, as shown in the bottom two rows in Fig. 6. But, when we had considered only the top 5 GO terms in each of the datasets, it was observed that SSc shared biological functions with Leukemia as well [Fig. 7]. In this experimental analysis, there has not associations between SSc and Lymphoma, probably that is because there is only one function (Cytokine-cytokine receptor interaction) shared between them which was revealed via pathway enrichment test. This also shows the importance of conducting both pathway enrichment and semantic similarities as they may reveal complementing evidence in deciphering the comorbid condition between SSc and cancer diseases.

### 3.5 Protein-protein interaction and hub protein identification

**Figure 8:**
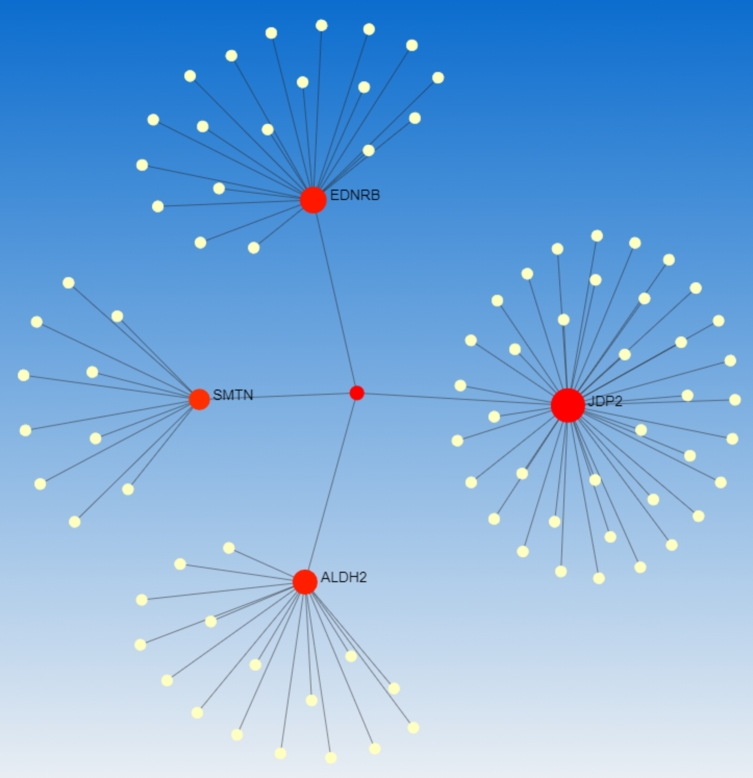
Protein-protein interactions to reveal hub proteins.

To identify hub proteins, at first, protein–protein interactions (PPI) network was constructed by retrieving the interaction of the common DEGs from STRING database as shown in Fig. 8. The PPI network consists of 88 nodes and 87 edges. This PPI analysis revealed four hub proteins, namely JDP2, EDNRB, ALDH2, and SMTN.

### 3.6 Literature support

To evaluate the validity of our work, we conducted an investigation of many literatures regarding the identified genes that have been clinically used as biomarkers for any of the three cancers. Table 3 shows the verified potential targets that confirm the validity of our study.

**Table 3:** Potential targets validation.

Gene name	Systemic Sclerosis	Lung cancer	Lymphoma	Leukemia
EDNRB	Fonseca et al. [55]	Wei et al. [56]	MacLean et al. [57]	Hsiao et al. [58]
ALDH2	Tan et al. [59]	Li et al. [60] Yang et al. [61]	-	-
CXCL2	Liang et al. [62]	Liang et al. [62] Keane et al. [63] Rivas-Fuentes et al. [64]	-	-
C1QB	Benbassat et al. [65]	Zhao et al. [66] Mangogna et al. [67]	-	-
CD93	Yanaba et al. [68]	Liu et al. [69]		Iwasaki et al. [70]
JDP2	-	Avraham et al. [71] Luo et al. [72]	Huang et al. [73]	Mansour et al. [74]
COL8A2	Dufour et al. [75]			
NAV3	-	-	-	Mansour et al. [74]
PKIB	-	Dou et al. [76]	-	-
RNPC3	Xu et al. [77]	-	Chen et al. [78]	Chen et al. [78]
SMOX	-	-	-	-

From Table 3, we observe that Fonseca et al. [55] identified EDNRB genes associated with Systemic Sclerosis, Wei et al. [56] identified EDNRB genes associated with Lung cancer and MacLean et al. [57] identified EDNRB genes associated with Lymphoma, Hsiao et al. [58] identified EDNRB genes associated with Leukemia. Tan et al. [59] identified ALDH2 genes associated with Systemic Sclerosis. Li et al. [60] Yang et al. [61] identified ALDH2 genes associated with lung cancer. Liang et al. [62] identified CXCL2 genes associated with Systemic Sclerosis. Liang et al. [62], Keane et al. [63] and Rivas-Fuentes et al. [64] identified CXCL2 genes associated lung cancer. Benbassat et al. [65] identified C1QB genes associated with Systemic Sclerosis. Zhao et al. [66] and Mangogna et al. [67] identified C1QB genes associated with lung cancer. Yanaba et al. [68] identified CD93 genes associated with Systemic Sclerosis. Liu et al. [69] identified CD93 genes associated with lung cancer. Iwasaki et al. [70] identified CD93 genes associated with Leukemia. Avraham et al. [71] and Luo et al. [72] identified JDP2 genes associated with lung cancer. Huang et al. [73] identified JDP2 genes associated with Lymphoma. Mansour et al. [74] identified JDP2 genes associated with Leukemia. Dufour et al. [75] identified COL8A2 genes associated with Systemic Sclerosis. Mansour et al. [74] identified NAV3 genes associated with Leukemia. Dou et al. [76] identified PKIB genes associated with lung cancer. Xu et al. [77] identified RNPC3 genes associated with Systemic Sclerosis. Chen et al. [78] identified RNPC3 genes associated with Lymphoma. Chen et al. [78] identified RNPC3 genes associated with Leukemia.

## 4 Discussion

The overall purpose of this research is to develop a bioinformatics and systems biology-based pipeline to retrieve novel knowledge from publicly available data repositories in terms of deciphering disease comorbidities. Our proposed pipeline, which is readily reproducible, may further be used for *omics* datasets related to any set diseases in order to find similarities between them. In this study, we have collected the high throughput sequencing data like: RNA-seq datasets from Gene Expression Omnibus (GEO) repository: (https://www.ncbi.nlm.nih.gov/geo) where we have identified comorbidities between SSc and any cancer, includes: Lung, Leukaemia, and Lymphoma.

In our proposed pipeline it has been hypothesized that SSc shows its comorbidities with cancer diseases for which relevant datasets were collected following our data-curation principle [see Methods] that includes both manual and automatic (using BioJupies) sample filtration based on GSM IDs. Although differential expression analysis of GEO datasets using linear models (i.e. limma R package), is assumed to have enough statistical power if the number of samples is equal or more than 3 for each group, but low number of samples added limitation to our pipeline in case of the outcomes at the end. Then we have applied GSEA that performed experiments with genome-wide expression profiles based on our designed case-control pair, and facilitated downstream analyses of SSc, particularly in terms of DEGs. Which discovered biological processes, molecular mechanisms, chromosomal position to portray the relations with other diseases. Then, the significance of the semantic similarity approach is observed, since it measured the similarity of separate disease (including different subtypes of same disease) depending on the chosen ontological terms yielded by other analysis, such as enrichment analysis of pathway and GO terms.

The semantic similarity methodology was used to calculate the relationship using identified pathologies involving important biomarkers and enriched GO term sets. The purpose of this study is to evaluate the associations between two diseases, thus the use of semantic similarities admittedly strengthened the detection and classification capability of unique biological processes engaged in each disorder/disease. To decipher and characterize the latent pathological details of comorbid condition between SSc and Lung cancer/Leukemia/Lymphoma, we have identified shared significant DEGs, molecular pathways, Gene Ontology, protein-protein interactions using enrichment or conditions based process. However, the semantic similarities among enriched GO terms revealed that SSc is highly correlated with Lung Cancer rather than Leukaemia and Lymphoma that also proved in other methods applied in our pipeline. Since our pipeline is data-driven, the study type and sample characteristics of the selected data played key roles in generating and validating our hypotheses. Although we have used two datasets for each of the diseases, we may claimed that various number of datasets collecting from different tissues or organs with at least three samples can be used to apply our pipeline for detecting comorbid condition between a pair of diseases. Both biologically and technically different data like RNA-seq or micro-array data can be used to reduce data-bias and offer robust inference. Since, this pipeline generates data-driven hypotheses that are biologically plausible (based on the experimental validations within the pipeline), experimenting with two or more diseases that are seemingly unrelated, would supposedly generate results accordingly. However, we argue that even experimenting with those ‘seemingly unrelated’ diseases using our data-driven pipeline paves the way to produce potential novel hypotheses – some of which may provide direction to further research.

Many of the previous approaches were designed for the disease comorbidity analysis by examining either single omics or clinical datasets. In [79] Elixhauser et al. [80], the medical comorbidity index, called the Charlson comorbidity file, was modified for using with clinical documents as well as analysed the International Classification of Diseases (ICD-9-CM) codes. Then the Charlson comorbidity index has been converted into a large number of ICD-9-CM codes. The Charlson Index Summation is thought to be relevant with this article, since the ICD-9-CM regulatory information used to assess the comorbidity relationship. In Hidalgo et al. [81], demonstrate a central phenotypic database namely Phenotypic Disease Network (PDN) has outlined the connections gained from previous experience of diagnosis of more than 30 million patients. The number of connections with a disease specified the number of comorbid diseases that acted as a risk factor for the patient diagnosed with the disease. Comorbidity4j introduced by Ronzano et al. [82], is an open-source Java platform for comprehensive comorbidity analysis. While an integrated Web interface is used to collect clinical input data and to customise the comorbid tests, then the results of those tests may be sorted against comorbidity indices and condition names, and analyzed through heat maps and network charts.

In our previous work [83], an R method was applied to identify the disease comorbidity using initial diagnosis, genetic and clinical data of a patient and also forecast the association between diseases which has developed using different packages (i.e. *pcalg*, *qtlnet*, etc). We [83] have also developed a framework to predict survival probability of a patient using survival analysis tools (e.g. *Net-Cox*, *rbsurv* etc). In this work, we have developed a R tool called “*POGO*” which integrates the multi-omics data, ontology term, and phenotypic information for more robust comorbidity prediction. But it doesn't count the genetic effect on diseases. In [84], a tool has been developed called *CytoCom* for Cytoscape. CytoCom is capable of clustering the Disease Comorbidity Network (DCN) based on the group of ICD9 disease codes that reveals the meaning of pathogenesis. Furthermore, contribute to the ultimately improvement of diagnosis and treatment analysis.

Many kinds of research have been conducted on molecular network-based approaches in order to help in drug development [85][86][87]. In earlier approaches, multi-omics, ontology, phenotypic information, and clinical data were used along with genetic data, but genetic effects on the diseases were not counted to demonstrate the relations. But two of the work used genetic effects by analyzing gene expression, molecular pathway, and Gene Ontology. A bioinformatics pipeline in R was developed [18][88] to evaluate gene expression, gene ontology (GO), and molecular pathways data by integrating Gene Set Enrichment Analysis and Semantic Similarity.

Our proposed approach to certify the comorbid conditions between SSc and cancer may be utilized by anyone, it has two basic applications that are discovered the potential pathways of SSc-related events that cause cancer disease progression and classify tentatively relevant health conditions by using omics and molecular evidence. With the advancement of such bioinformatics analysis, it may offer new opportunities for physicians to make a decision, such as potential danger evaluations, cancer detection, and subtyping, drug treatment, and dosage selection, which is a step towards the development of genuinely regenerative medicine [89][90]. The approach will then offer profound fresh perspectives into disease mechanisms, and those defined disease mechanisms may be helpful to define promising pharmacological strategies for further studies.

## 5 Conclusions

The aim of this manuscript was to identify pathways of SSc patients that play a vital role in developing different cancer, and also draw potential therapeutic drug targets (biomarkers) by utilising and analysing transcriptomics data, molecular pathways, protein-protein interactions and identification of hub proteins along with semantic similarity in terms of DEGs and GO pathways. Hence the methodology has identified required evidence to verify the linkage between Systemic Sclerosis and selected cancers. In addition to cancer diseases, the proposed strategy could be generalized mostly as a comorbidity chart by adding certain disease details. In terms of basic biological functions, pathways as well as omics info, such as GO, we have found that certain cancer conditions were strongly related to SSc. Our results suggest that by utilizing bioinformatics techniques, the evolution of emerging diseases may be detected and analyzed as it provides the opportunity to develop an understanding of various diseases functions. There is a growing interest among research communities in understanding comorbidity associations since it might uncover new knowledge concerning disease-causing facts as well as potential therapeutic strategic objectives. In exposing potential disease associations and prospects for drug development, this research illustrates the importance of an advanced bioinformatics and system biology-based approach. We have also assumed that such a form of methodology would be useful for providing evidence-based decisions on comorbid conditions. By combining other disease information alongside various types of cancer, our suggested approach may be applied as an association between various conditions map. It may be also used by researchers and healthcare professionals as an essential method to discover the specified fundamental disease's processes that underpin the nature and pathology of comorbid conditions and to develop more reliable and efficient therapies, possibly in a highly personalized and customized pharmacotherapic framework.

## Declarations

### Author contribution statement

Md Khairul Islam: Conceived and designed the experiments; Performed the experiments; Analyzed and interpreted the data; Contributed reagents, materials, analysis tools or data; Wrote the paper.

Md Rakibul Islam: Analyzed and interpreted the data; Wrote the paper.

Md Zahidul Islam: Conceived and designed the experiments; Performed the experiments; Wrote the paper.

Md. Habibur Rahman: Performed the experiments; Analyzed and interpreted the data; Wrote the paper.

Md Mainul Islam Mamun: Contributed reagents, materials, analysis tools or data.

AKM Azad: Contributed reagents, materials, analysis tools or data; Wrote the paper.

Mohammad Ali Moni: Conceived and designed the experiments; Analyzed and interpreted the data; Contributed reagents, materials, analysis tools or data; Wrote the paper.

### Funding statement

This research did not receive any specific grant from funding agencies in the public, commercial, or not-for-profit sectors.

### Data availability statement

Data associated with this study has been deposited at https://www.ncbi.nlm.nih.gov/gds

### Declaration of interests statement

The authors declare no conflict of interest.

### Additional information

No additional information is available for this paper.
